# Prevalence of patients at risk for developing obstructive sleep apnea based on the STOP‐BANG questionnaire at King Abdulaziz University Faculty of Dentistry

**DOI:** 10.1002/cre2.688

**Published:** 2022-11-09

**Authors:** Maisa O. Al‐Sebaei, Mohamed S. Bamashmous, Mohamed H. Mirdad, Mohammed A. Sindi

**Affiliations:** ^1^ Department of Oral and Maxillofacial Surgery, Faculty of Dentistry King Abdulaziz University Jeddah Saudi Arabia; ^2^ Department of Dental Public Health, Faculty of Dentistry King Abdulaziz University Jeddah Saudi Arabia; ^3^ Ministry of Health Jeddah Saudi Arabia

**Keywords:** early detection, OSA, prevalence, risk assessment, STOP‐BANG

## Abstract

**Objective:**

Obstructive sleep apnea (OSA) is a common sleep disorder. Its susceptibility can easily be detected when it is at an early stage as can patients who are at risk of OSA. A simple questionnaire such as STOP‐BANG (SB) can facilitate early detection. Our study aims to assess the risk of OSA and evaluate its correlating risk factors in patients attending King Abdulaziz University Faculty of Dentistry (KAUFD), Jeddah, Saudi Arabia, using the SB questionnaire.

**Methods:**

Ethical approval was obtained. A random sample of patients ages 18–80 years, who visited KAUFD between November 2016 and April 2017 were recruited. Three highly trained and calibrated dental interns obtained the following measurements (weight, height, blood pressure, and neck circumference) and administered the questionnaire in a face‐to‐face interview.

**Results:**

A total of 55 patients (62% females) participated in the study with a mean age of 34.6 years and hypertension reported as 16.4%. According to the SB, 31% of patients were found at high risk of OSA. Large neck circumference (40.22[±4.7] cm) and gender (66% males) were found to be significantly correlated with high‐risk patients; *p* < .001.

**Conclusion:**

Approximately one third of the patients were at high risk of OSA, with men and patients having large neck circumference being significantly more affected. Systolic blood pressure, hypertension, and smoking were found to be high in high‐risk patients. However, they were not statistically and significantly different from those patients who were not in high risk. The role of a dentist should be to utilize the questionnaire to detect any patient at risk of OSA and refer them accordingly.

## 1 BACKGROUND

Obstructive sleep apnea (OSA) is one of the most common sleep disorders in the world. It can be characterized by one or more breath stopping while asleep. In OSA, breathing may stop for 10 s at least five times in 1 h. These stops are considered hypopneas if your oxygen intake is decreased, and apneas if it completely stops (American Academy of Sleep Medicine, 1999; Johns Hopkins Medicine, 2022). The signs and symptoms are caused by the repeated collapse of the upper airway. The main symptoms are usually loud snoring, fatigue, and daytime sleepiness. Studies show that there is a link between obesity and hypertension as well as a link between OSA and hypertension (Kannel et al., 1967). There is an increased risk for hypertension in patients with untreated OSA (Marin et al., 2012; Silverberg et al., 1998). Obesity is considered a major risk factor for OSA as it causes upper airway obstruction due to alteration in the anatomical structures (BaHammam et al., 2008; Punjabi, 2008; Young et al., 1993). Other symptoms may include morning headache, restless sleep, low energy, and less concentration. Bed partners are usually the ones to prompt the patient to seek medical attention (Lam et al., 2010). Despite advances in diagnostic technology, sleep apnea is still unrecognized by many, worldwide, and leads to decreased work performance, excessive daytime sleepiness, and nonfatal or fatal car accidents causing avoidable fatality if diagnosed early (Leung & Douglas Bradley, 2001; Marin et al., 2005; Rodenstein, 2009; Wolk et al., 2003). Polysomnography is the gold standard method for diagnosing sleep apnea, but it is also accompanied by many limitations, such as, requiring an overnight stay of the patient, personnel monitoring of complex physiologic data, being time‐consuming, and not readily available everywhere. Due to its nature and the duration it takes, it is usually performed on patients who are suspected to have OSA. To identify those patients, there are multiple screening tools to collect related information that could then be used to give the patient a score. The score can help determine the likelihood of the patient having OSA. One of the most famous tools is the STOP‐BANG (SB) questionnaire. These screening tools were validated even though they do not replace polysomnography, they are still an excellent preoperative screening method (Lapin et al., 2018; Nagappa et al., 2015; Netzer et al., 1999).

Dental practitioners can have a role in the early detection of OSA and subsequent interventions and treatment. The use of a screening tool such as SB can facilitate early detection and can be used in healthcare centers such as dental clinics, in addition to sleep clinics. Later on, orthodontists and prosthodontists can become involved in the treatment of OSA by prescribing and fitting appliances. Oral and Maxillofacial surgeons may also be involved and apart from performing surgery, may prescribe breathing aids such as CPAP (Ng & Yow, 2019; Riley & Powell, 1990).

The SB questionnaire was first developed in 2008. It is a straightforward, easy, and reproducible questionnaire that is used as a screening tool to detect high‐risk patients for OSA, and it has been tested for its validity and reliability (Chung et al., 2008; Nagappa et al., 2015).

The prevalence of OSA in multiple countries is 3%–7% for adult men and 2%–5% for women. Disease prevalence was higher in different subsets of the population such as overweight, old people, and minority races (Nagappa et al., 2015). In Saudi Arabia, few studies were conducted to identify the prevalence of risks and symptoms of sleep apnea. They have been conducted in regions in the middle of the country. BaHammam et al. in 2008 found that 33.3% of middle‐aged men with a mean age of 45 years were considered at risk for OSA (Netzer et al., 1999). The same research group conducted another study on middle‐aged women and showed that 39% were at risk for OSA (Wali et al., 2015). There are few other studies that measured the correlation between OSA and other systemic conditions, but used the SB questionnaire, such as the study published by Elnour et al. in 2019 that found a negative correlation with coffee intake (Elnour et al., 2019), Felfeli et al. in 2020 that found a positive correlation with retinal vein occlusion (Felfeli et al., 2020), Kalakattawi et al. in 2017 that found a positive correlation with type II diabetes (Kalakattawi et al., 2017), and Wali et al. in 2015 that found a positive correlation with coronary artery disease (Wali et al., 2015). We could not find suggestions regarding how we can apply the criteria of patients at high risk of OSA in our region.

All of the previous studies were conducted over a large geographic area. Low sample size was always an issue due to the nature of the condition. They also restricted the samples to a single gender.

The aim of this study was (1) to assess the prevalence of patients who were at risk for developing OSA among a population of patients seeking treatment at the faculty of dentistry at King Abdulaziz University, through the SB screening tool and (2) to assess the factors (gender, BMI, BP, and neck circumference) associated with the individuals who are at high risk for OSA.

## 2 MATERIALS AND METHODS

This study proposal was approved by the research ethics committee at the Faculty of Dentistry, King Abdulaziz University (REC‐KAUFD) approval number (043‐15).

### 2.1 Study design and participants

This was a cross‐sectional study. We recruited the study population randomly from walk‐in patients seeking various dental treatments from the screening clinics of the University Dental Hospital at King Abdulaziz University patients. Selected subjects were between 18 and 80 years old and were willing to participate in the study. We collected the sample between November 2016 and April 2017. Patients signed a consent and were informed about their voluntary right to participate. They were reassured that their anonymity would be protected and that all data collected from them would be treated as confidential.

### 2.2 Data collection

We collected the following demographics for each participant: age, gender, and smoking habits. We used electronic means in the collection of some of the parameters (weight, height, blood pressure, and neck circumference). We administered the SB questionnaire by a trained dental intern using a face‐to‐face interview process following the original SB questionnaire.

### 2.3 Questionnaire

The original, well‐validated sleep apnea questionnaire, SB, was used (Chung et al., 2008). The scores of this questionnaire range from 0 to 8. It contains four subjective dichotomous (yes/no) questions about snoring, tiredness, observed breathing cessation or choking during sleep, and if the patient is being treated for hypertension. The questionnaire also contains four demographic data questions that are scored based on their category. The demographic questions are the body mass index (BMI), age, neck circumference, and gender. For the general population, scoring is divided into three sections: low risk for OSA (yes to 0–2 questions), intermediate risk of OSA (yes to 3–4 questions), and high risk of OSA (either yes to 5–8 questions, or yes to 2 or more of the four STOP questions with the additions of either Male gender, BMI above 35 kg/m^2^, or neck circumference of 43 cm in males and 41 cm in females; BaHammam et al., 2008; Elnour et al., 2019).

### 2.4 Data management

We calculated the BMI based on the height and weight of the patients. We measured the patients' neck circumference in cm using a flexible measuring tape. We also used a digital blood pressure monitor (HEM‐907XL, Omron Healthcare, Illinois, USA) to record blood pressure. We classified the patients into two groups, a normal and elevated blood pressure group that included patients with a systolic reading of <130 mmHg and diastolic of <80 mmHg, and a high blood pressure group that included patients with stage 1 hypertension or above (2017 Classification, American Heart Association/American College of Cardiology). This was carried out to identify the group in need of urgent care. Finally, we divided patients according to the SB classification into 2 groups: patients who were classified to be at a low or intermediate risk of OSA were grouped together, and patients who were classified to be at a high risk were in a separete group; this was in order to identify patients who may require urgent referral to a specialist at a sleep medicine center.

### 2.5 Data analysis

We performed the data coding and analysis using the International Business Machine Corporation's Statistical Package for the Social Sciences for Windows, version 26.0 (IBM Corp., Armonk, N.Y., USA). We performed a descriptive statistic to define the characteristics of the study with mean (SD) and frequency/percentages where it was appropriate. We used the Chi‐square and *t*‐test to test the association of SB risk classification with different factors using α *p* < .05 for the significance level.

We also described each element of the SB questionnaire using frequencies and percentages of the participants’ answers.

## 3 RESULTS

A total of fifty‐five patients were enrolled in this study, aged between 18 and 78 years old, with a mean age of 34.62(±14.9) years, of which 34 (61.8%) are females. The SB score of the participants ranged from 0 to 7, with the mean score being 2.29(±2). Additional demographic and health information can be found in Table 1. Two of the male participants were presented with neck circumference greater than 43 cm, while two of the females presented neck circumference greater than 41 cm. Answers of the participants to the STOP section of the questionnaire can be found in Table 2.

**Table 1:** Descriptive statistics for demographics and health variables

Parameters	*N*	Min	Max	Mean	SD
BMI (kg/m^2^)	55	15.24	45.73	25.42	5.9
Neck circumference (cm)	55	25.00	47.00	35.80	5.3
Blood pressure systolic (mmHg)	54	98	168	128.09	16.2
Blood pressure diastolic (mmHg)	54	62	103	78.98	13.2
STOP‐Bang score	55	0	7	2.29	2.0

**Parameter**		**Freq (55)**		%	
Gender	Male	21		38.2	
	Female	34		61.8	
Do you smoke?	No	41		74.5	
	Yes	14		25.5	
STOP‐Bang score category	Low + intermediate risk of OSA	38		69.1	
	High risk of OSA	17		30.9	
Hypertension	No	46		83.6	
	Yes	9		16.4	
Diabetes	No	48		87.3	
	Yes	7		12.7	
Fit and healthy	No	39		70.9	
	Yes	16		29.1	
None	No	35		63.6	
	Yes	20		36.4	
Others	No	46		83.6	
	Yes	9		16.4	

Abbreviations: BMI, body mass index; OSA, obstructive sleep apnea.

**Table 2:** Descriptive statistics of the STOP‐BANG scale

Parameter		Freq (55)	%
Do you SNORE loudly (louder than talking or loud enough to be heard through closed doors)?	No	44	80.0
	Yes	11	20.0
Do you often feel TIRED, fatigued, or sleepy during daytime?	No	20	36.4
	Yes	35	63.6
Has anyone OBSERVED you stop breathing during your sleep?	No	48	87.3
	Yes	7	12.7
Body Mass Index more than 35 kg/m^2^?	No	51	92.7
	Yes	4	7.3
Do you have or are being treated for High Blood Pressure?	No	42	67.4
	Yes	9	16.4
	Don't know	4	7.3

In bivariate analyses, we found that gender and neck circumference were statistically significant predictor of SB categories at α *p* < .001. We found that gender (66.7% of males vs. 8.8% of females) and higher neck circumference are in the high‐risk group. Both BMI and smoking were not statistically significant predictors at α *p* > .05. For blood pressure, only the systolic blood pressure category showed statistically significant association *p* < .004 (Table 3).

**Table 3:** Bivariate *t*‐test and chi‐square between STOP‐BANG and the different variables

Variables	Total (55)	STOP‐Bang score category		
		Low + intermediate risk of OSA 38 (69.1%)	High risk of OSA 17 (30.9%)	*p* Value
Age	34.62 (14.85)	32.39 (12.69)	39.59 (18.26)	.15
Neck circumference	35.80 (5.32)	33.82 (4.32)	40.22 (4.7)	<.001^a^
**Gender**				
Male	21	7 (33.3%)	14 (66.7%)	<.001^b^
Female	34	31 (91.2%)	3 (8.8%)	
**Do you smoke?**				
Yes	14	7 (50%)	7 (50%)	.07
No	41	31 (75.6%)	10 (24%)	
**BMI category**				
Underweight	6	5 (83.3%)	1 (16.7%)	.21
Normal	23	18 (78.3%)	5 (21.7%)	
Overweight and obese	26	15 (57.7%)	11 (42.3%)	
**Blood pressure systolic category**				
Normal and elevated	35	29 (82.9%)	6 (17.1%)	.004^b^
High	20	9 (45%)	11 (55%)	
**Blood pressure diastolic category**				
Normal	27	21 (77.8%)	6 (22.2%)	.17
High	28	17 (60.7%)	11 (39.3%)	
**Blood pressure category**				
Normal	22	18 (81.8%)	4 (18.2%)	.09
High	33	20 (60.6%0	13 (39.4%)	

Abbreviation: OSA, obstructive sleep apnea.

^a^ Significant using *t‐*test @<.05 level.

^b^ Significant using Chi‐square test @<.05 level.

We built a logistic regression model using SB categories as our outcome measure and we included gender neck circumference and systolic blood pressure as our predictors. Our global model hypothesis was statistically significant at α *p* < .05. We found that male gender and high neck circumference stayed as statistically significant predictors for the SB categories controlling for the other confounding variables in the model at α *p* < .05 (Table 4).

**Table 4:** Logistic regression model predicting high risk from different factors

		Odds ratio	95% CI for odds		*p* Value
Controlled for variables		Lower	Upper		
Neck circumference		1.290	1.035	1.608	0.023
Gender (male)		12.830	2.380	69.168	0.003
Systolic blood pressure (normal)		1.295	0.184	9.115	0.795
Constant		0.000			0.012

Abbreviation: CI, confidence interval.

## 4 DISCUSSION

In this study, we address the risk of sleep apnea in dental patients through the validated SB questionnaire. Although it was conducted in one dental institute, it gives a general idea of patients at high risk among our dental population.

The prevalence of high‐risk patients with OSA according to SB was 31%. Higher risk of OSA affected males more than females. This is consistent with the results of previous studies that men were more liable to be at risk of OSA than women (Lee et al., 2008; Nagappa et al., 2015; Wali et al., 2017). This could be contributed to the variation in upper airway anatomy, hormones, and aging between both genders (Lin et al., 2008).

Many epidemiological studies identified weight as the main risk factor for OSA. Other studies concluded and confirmed the association that weight is an independent risk factor as it alters the airway mechanism by constricting it from the increase of fat deposition. This makes neck circumference larger, causing instability in the respiratory system (Fritscher et al., 2007; Peppard et al., 2000; Wolk & Somers, 2006). In the SB questionnaire, it shows that overweight and obese represented 20% considerably high compared to normal weighted and underweighted patients that shows only 9.1% and 1.8% at risk for OSA, respectively (Kannel et al., 1967; Nagappa et al., 2015).

In our sample, 30.9% were at high risk for OSA, which is consistent with similar studies in other parts of Saudi Arabia (Bahammam et al., 2009; Netzer et al., 1999). Neck circumference, BMI, and blood pressure were all found to be higher in those individuals with a higher risk of OSA, but only the gender, neck circumference, and systolic blood pressure were statistically significant, and after performing logistic regression, only gender was statistically significant.

Additional multicenter studies are needed. More studies are needed that look at diagnosed OSA patients to establish the efficiency of this tool and its significance in our population. Since neck circumference differs from one ethnicity to another, studies where ethnicity is controlled for are needed. Larger studies involving samples of patients are needed in our population. This is important in our population specially in our community's widespread hypertension, which is considered a well‐documented risk factor (Hou et al., 2018).

In conclusion, approximately one third (30.9%) of our sample are considered at high risk for OSA. Men were considered at high risk more than women. The dental practitioner can play an important role in the early identification of patients at high risk for OSA, especially when dental patients may exhibit craniofacial abnormalities. This is important for early screening and prompts referral to sleep specialists at an early stage. The parameters required to screen such patients are simple and readily available in most dental offices, especially dental hospitals or teaching institutions. Since there is a large number of patients in such institutions, it is recommended that identifying, screening, and referring become a routine practices to avoid serious complications.

## AUTHOR CONTRIBUTIONS

Mohamed H. Mirdad and Maisa O. Al‐Sebaei conceived the idea; Mohamed H. Mirdad collected the data; Mohamed S. Bamashmous and Mohammed A. Sindi analyzed the data; Mohammed H. Mirdad and Mohammed A. Sindi wrote the manuscript; and Maisa O. Al‐Sebaei and Mohamed S. Bamashmous revised the manuscript.

## CONFLICT OF INTEREST

The authors declare no conflict of interest.

## Data Availability

Due to patient confidentiality, data are available from the corresponding author upon reasonable request.
